# Transferability of Technical Skills Across Robotic Surgery Platforms: A Scoping Review

**DOI:** 10.7759/cureus.56429

**Published:** 2024-03-19

**Authors:** Michael Devine, Marie Morris, Dara Kavanagh

**Affiliations:** 1 Department of Surgical Affairs, Royal College of Surgeons in Ireland/Hermitage Medical Clinic, Dublin, IRL; 2 Department of Surgical Affairs, Royal College of Surgeons in Ireland, Dublin, IRL

**Keywords:** robotic surgery curriculum, multi-platform, skill transfer, competency based medical education, robotic surgery education

## Abstract

As the application of robotic approaches to surgery continues to broaden, new consoles have been introduced to the market. Due to the global utilization of a single platform, previously validated curricula have not been assessed on new robotic systems. Surgery by its nature occurs in a high-stakes environment, potentially exacerbated by non-standardized robotic systems. The aim of this review is to critique the evidence available regarding the transferability of technical skills across robotic platforms.

A scoping review utilizing the Medline (Pubmed) and Cochrane Databases was conducted. Full texts were reviewed and appraised. Selected articles were eligible for inclusion if they investigated the ability or implications of the transfer of skill across robotic platforms. Data was extracted, coded inductively, and themes synthesized. NVIVO software was used as an adjunct for this qualitative analysis.

Following the removal of duplicates a total of 278 papers were screened according to the eligibility criteria. Fifty full-text articles were reviewed and four met the criterion for inclusion. Novices’ performance across platforms was comparable. Increasing levels of prior robotic experience revealed an improvement in technical performance on a novel robotic platform. Safety metrics appear comparable across systems.

Quantifying learning curves across robotic platforms and their implications for the robotic surgeon in training remains to be determined. Future research needs to address the gaps in the literature by clearly defining the extent of technical skills transfer between robotic platforms. These factors will guide the next iteration of surgical training curriculums and regulations for robotic surgery.

## Introduction and background

Robotic surgery and platform evolution

A robot may be defined as a technological system capable of performing specific tasks automatically, according to a fixed or modifiable program [1]. Robotic surgery was introduced to clinical practice in the 1980s, performing neurosurgical biopsies and prostatectomies. These robotic systems were programmed pre-operatively with radiological images and undertook a procedure in an automated fashion [2,3]. Telemanipulation and telepresence were combined to develop the contemporary iterations of master-slave robotic surgical systems (Table 1) [1,4,5].

**Table 1:** Definitions of common terms relating to robotic surgery

Term	Definition
Robot	A technological system capable of performing specific tasks automatically, according to a fixed or modifiable program [1]
Transfer	The application of a skill or competence from one domain or context to another
Skill	The ability, proficiency or dexterity to carry out tasks that come from education, training, practice or experience [4]
Competence	A combination of knowledge, skills and attitudes appropriate to the context
Telepresence	Immersion of the human into a remote environment. Through a multimodal human-machine interface the human perceives and acts as in the real world [5]
Telesurgery	Operating on a patient from a geographically remote location, displaced from the operating table
Telemanipulation	Control of a tele-operated device on a force and motion level [5]

The Food and Drugs Administration (FDA) approved the Da Vinci 2000TM robotic system (Intuitive Surgical, Sunnyvale, CA, SA) in 1999. The adoption of robotic surgery has undergone exponential growth since then. In the past decade alone, between an eight- and 22-fold increase in robotically performed surgeries has been documented [6,7]. Proponents for the adoption of robotic surgery highlight benefits to the patient including reduced conversion rates to open [8], decreased blood loss, reduced length of hospital stay [9], and improved patient-reported outcome measures [10]. Advantages to the surgeon include a more comfortable operating position, avoidance of fatigue, a stable 3D platform, improved anatomic visibility, and increased ergonomic access to confined operative fields. Disadvantages include the capital involved in purchasing the robotic system, increased operating time, and an additional learning curve to be mastered [11].

**Figure 1:**
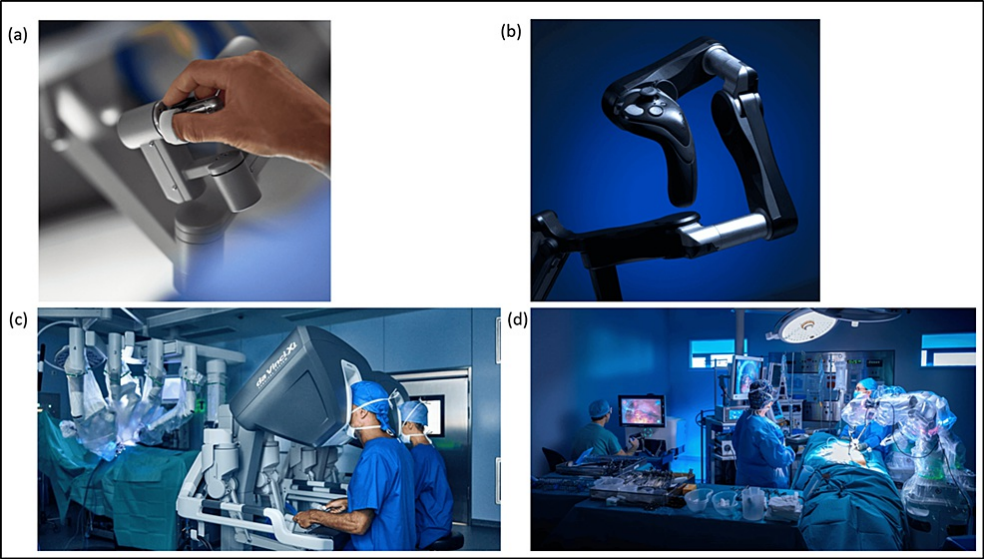
Differences in control design of robotic platforms: (a) Da Vinci Xi, (b) Versius. Differences of robotic system configuration/telemanipulation: (c) Da Vinci Xi, (d) Versius. Reproduced with permission from Intuitive Surgical, and Cambridge Robotic Medical, respectively.

Over 1,800,000 robotic surgery cases are carried out per annum, with almost 8,000 robotic consoles installed in healthcare institutions worldwide [12]. The expiration of Intuitive’s patents has allowed an opportunity for other vendors to enter the market. The continued increase in demand by patients and clinicians has led to the further introduction of new systems. Each robotic platform has different operational structures, and configurations of design (Figures 1a-1d, Table 2) [13,14]. Differences are due to necessity and design, to avoid copyright and to aid operational control. Further innovations and technologies to augment robotic surgery are in various stages of development including the Taurus III (Verb Surgical Inc, CA, USA), Enos (Titan Medical Inc, Ontario, Canada), and Raven II systems [14]. Thus far, national and international regulatory bodies have approved 12 robotic platforms for clinical use [15].

**Table 2:** Variation in available robotic surgical platforms

Platform	Arm Carts	Degrees of Movement	Haptic Feedback	Console Design	Device Control	3D	Tracking	Year Approved
da Vinci Xi	Single	Seven	No	Closed	Finger loops	Yes	No	2014
Senhance	Multiple, up to four	Seven	Yes	Open	Laparoscopic handles	Yes	Eye tracking	2016
RobOtol	Single	Seven	No	Open	Tactile pad	2D	No	2016
Revo-I	Single	Seven	No	Closed	Finger loops	Yes	No	2017
da Vinci SP	Single	Seven	No	Closed	Finger loops	Yes	No	2018
Versius	Multiple, up to four	Seven	Yes	Open	Joystick handles	Yes	No	2019
Avatera	Single	Seven	No	Closed	Finger loops	Yes	No	2019
Hinotori	Single	Eight	No	Closed	Finger loops	Yes	No	2020
Dexter	Multiple, three	Seven	Yes	Open	Joystick handles	Yes	No	2020
Hugo RAS	Multiple, up to four	Seven	No	Open	Pistol grip	Yes	Head tracking	2022
Mantra	Multiple, up to five	Seven	No	Open	Joystick handles	Yes	Head tracking	2023
MIRA	Miniature single system	Seven	No	Open	Pistol Grip	Yes	No	2024

Robotic Surgery Training Curriculums and Their Transfer Across Platforms

There is no standardized postgraduate accredited curriculum for robotic surgery [16]. Due to the industry monopoly by one robotic system to date, the single institute robotic surgical curricula which do exist are not validated on a multi-platform basis. It remains to be elucidated if a generic robotic curriculum can be developed to train residents to competency independent of the robotic platform used.

Aim

The aim of this review is to assess the published research evidence regarding the transferability of technical skills from one robotic system to another.

## Review

Methods

Protocol

As our aim was to identify knowledge gaps [17], a scoping review was deemed the most appropriate approach. This scoping review was undertaken and reported in accordance with the Preferred Items for the Reporting of Systematic Reviews and Meta-Analyses (PRISMA-ScR) guidelines 2018 (Scoping Review extension) [18]. A protocol was developed in accordance with these guidelines [19].

All original research articles, abstracts, and conference proceedings from database inception to present; or any grey literature identified or referenced were eligible for inclusion. Articles not published in English were not excluded if key information was presented in English within the abstract or main text

Search Strategy

The Medline (Pubmed) and Cochrane Databases were explored. The final search was carried out on January 1, 2024. The search was carried out using the following terms: (transfer OR transferability OR transference) AND (robot OR robotic) AND surgery AND (skill OR competency OR proficiency OR expert OR novice OR intermediate OR master OR mastery). No limits were applied. 

Titles, abstracts, and keywords were screened for relevance by the author (MD). Full texts were reviewed and appraised. Selected articles were eligible for inclusion if they studied the ability to, or examined the implications of transfer of technical skill across robotic platforms (Table 3). The reference lists of included articles were also examined using the same screening and eligibility criteria. Duplicate results were removed.

**Table 3:** Inclusion and exclusion criteria

Inclusion Criteria	Exclusion Criteria
Randomised controlled trials, cohort studies, case–control studies, other quasi-experimental studies	Articles wholly not published in English
White or grey literature from surgical colleges or industry	Construct validation of curriculums or trainee views when dealing with a solitary robotic console
Data pertaining to performance of participants across two or more robotic systems	Data assessing the transferability of competency in open/laparoscopic surgery to a single robotic system
Performance as tested in simulation, dry/wet lab, or in vivo	Data pertaining to competency degradation or learning curve appraisal across just one robotic platform
Either/both technical skills competency or learning curve	

Data Extraction and Analysis

Data extracted from the articles included: the robotic systems utilized, skills assessed, interface used for assessment (simulation program, dry/wet lab, in vivo), proficiency level of participant, and competency on transfer to operation of another system.

Data was extracted and grouped into themes using content analysis [20]. An inductive extraction process was utilized. Open coding was employed to develop a coding framework. This involved extracting data of interest from the included articles and transferring these to a worksheet for coding analysis. Subsequent categorization into nodes and emergent themes was conducted by the author (MD). NVivo software was used to augment this qualitative process. Discussion amongst the authors (MD, MM, and DK) was included to resolve any difficulty with coding the data.

Results

Articles

**Figure 2:**
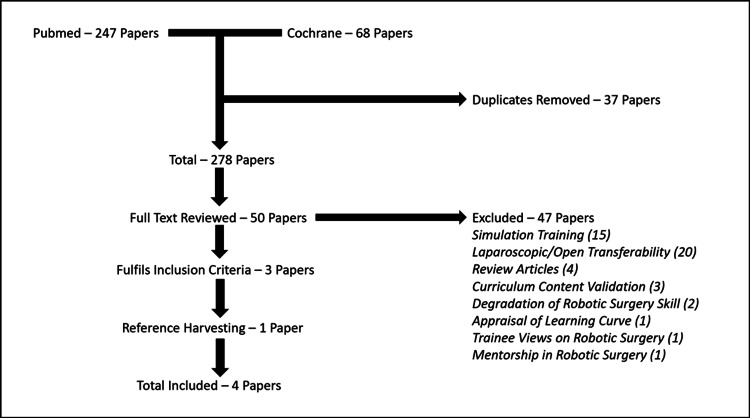
Search strategy and selection process.

Following removal of duplicates a total of 278 papers were screened according to the eligibility criteria. Fifty full-text articles were reviewed, and three studies were eligible for inclusion. Through manual reference harvesting one further paper was identified (Figure 2) [21-24].

Study Demographics, Designs, and Outcomes Measured

Studies ranged in size from 10 to 71 participants (Table 4) [21-24]. Experience ranged broadly. The largest study assessed 71 participants on the HUGO RAS (Medtronic, Minneapolis, MN, USA) simulator, 10 of which were robotic surgeons. A robotic surgeon was defined as a surgeon who had performed a minimum of 5 operations on the Da Vinci platform. Only 6 of the remaining 61 participants had any surgical experience, with the remainder accounted for by students [22]. A comparative study assessing Da Vinci's multi-port (MP) performance with subsequent single-port (SP) performance recruited 15 surgeons. Even distribution occurred amongst novices naïve to robotic surgery, robotic surgeons with extensive MP experience (>250 cases), and robotic surgeons with extensive MP (>250 cases) and SP experience (>50 single port cases) [24]. In the validation of Versius’ (Cambridge Robotic Medical, Cambridge, United Kingdom) training program, eight of the 17 recruited surgeons had at least some robotic experience (>5 robotic operations performed), and a further five surgeons were deemed expert Da Vinci users (>30 procedures performed) [23]. The non-randomized, matched, cross-over study examining performance on the Da Vinci training simulator and the HUGO RAS simulator recruited 10 residents and fellows. They allocated five to each group and anonymized the consoles when publishing their results. Complete data was collected on seven participants for console A and eight on console B. The prior robotic experience of the total recruited participants is stated, however, if differences exist in those participants used for analysis is not [21].

**Table 4:** Characteristics of included articles

Authors	Year	Origin	Population	Speciality	Size	Methods	Outcome	Robotic Platform
Ghazi et al. [24]	2023	United States	Attendants Residents	Urology	15	Dry lab using hydrogel model assessing technical skill (anastomosis), Survey	Competency, Task Load, Participant Perspectives	Da Vinci Xi/Si, Da Vinci SP
Sighinolfi et al. [22]	2023	Italy	Attendants, Residents, Medical & Nursing students	All surgical specialities	71	Simulation modules assessing technical skill (pick + place)	Competency	Hugo RAS (Prior experience Da Vinci)
Larkins et al. [21]	2022	Australia	Residents Fellows	General Surgery & Urology	10	Simulation modules assessing technical skill (4 x modules), Survey	Competency, Safety, Trainee Perspectives	Da Vinci, Hugo RAS
Butterworth et al. [23]	2021	United States	Attendants	General Surgery, Obstetrics & Gynaecology, Urology	17	Wet lab using cadavers	Competency	Versius (Prior experience Da Vinci)

Performance was determined by virtual reality simulation assessments (Mimic Technologies, Surgical Science, Sweden) [21,22], dry laboratory performance of a ureterovesical anastomosis (UVA) [24], and a wet laboratory environment with cadavers undergoing specialty-specific procedures [23].

Outcomes of transferable skills assessed included competency, safety metrics, trainee perception, and cognitive task loads (Tables 4, 5) [21-24]. Global Evaluative Assessment of Robotic Skills (GEARS) [23,24], Robotic Anastomosis Competency Evaluation (RACE) [24], time to completion, and kinematic data were used to distinguish performance across platforms [21,22].

**Table 5:** Key results from papers

Authors	Year	Key Findings of Evidence
Ghazi et al. [24]	2023	Surgeons with mastery of the multi-port system, regardless of single port experience performed better than novices. Multi-port experts had reduced performance compared to those with previous single-port use. Experts perceived difficulty ratings were comparable with novices, despite higher performance scores. Performance and cognitive load was similar for both platforms in novices.
Sighinolfi et al. [22]	2023	Robotic surgeons (with previous Da Vinci experience) performed better than laparoscopic surgeons and naïve participants on the Hugo RAS console (P=0.004, P=0.002).
Larkins et al. [21]	2022	Non-statistically significant trend towards reduced time to pass in console B versus console A (slower as 1^st^ console in ‘Matchboard’ and as 2^nd^ console in ‘Thread the Rings 2’ simulations). No difference in safety metrics. Participants felt there was good overlap of skills across platforms.
Butterworth et al. [23]	2021	Improvement in Versius performance metrics most notable in surgeons with extensive Da Vinci experience, moving from intermediate at initial assessment to expert competency at end of training program.

Though all study participants underwent orientation before assessing performance on the novel robotic system, the process was not standardized. A brief tutorial and unstructured practice were used in two studies [21,22]. Participants were allowed to perform a self-directed practice assessment with no feedback in the UVA study [24], while formal familiarization and training took place as part of the Versius education validation study [23].

Transfer of Technical Skills Across Robotic Platforms

Comparing performance directly between the Da Vinci and HUGO RAS simulators no statistically significant difference in times to pass or proficiency was observed [21]. Console A performance was improved when it was the second console a participant used as opposed to the first console. Overall times on console B were noted to be quicker than on console A independent of the order of use. Cumulative exposure did not lead to improved performance in the final task or associated kinematic metrics, though this may represent fatigue of participants [21].

Robotic surgeons showed a statistically significant decreased time to exercise completion in comparison to novices and laparoscopic surgeons. However, experts and novices had similar kinematic data pertaining to the economy of motion and master workspace usage [22].

In all assessments of the Versius training program, robotic surgeons with extensive experience consistently outperformed the minimal robotic experience cohort and novices. The margin of improvement throughout the four days was greater than the other two groups (mean improvement of GEARS assessment of 4.3 for experts, mean improvement of 1.5 for some robotic experience and mean improvement of 2.7 for novices). Initial performance using the GEARS assessment for robotic surgeons with limited experience was superior to novices. Performance for both improved to a similar level by the validation assessment. In the novice cohort, the GEARS domain of autonomy was the only area not to achieve an intermediate level on the validation assessment [23].

Novices to robotic surgery performed equally across platforms in the Da Vinci MP and SP systems (GEARS assessment of 17.3 (MP) and 18.1 (SP)) [24]. Expert MP surgeons with no SP experience performed superiorly on the MP system (GEARS 27 & RACE 26.9 MP vs GEARS 24.1 & RACE 21.8 SP, p-value < 0.05). Expert MP and SP surgeons had a superior performance on the SP platform (GEARS 27 & RACE 25.7 MP vs GEARS 25 & RACE 23.7 SP, p-value > 0.05) [24]. Expert MP users with SP naivety effectively transferred skills in the domains of depth perception, bimanual dexterity, efficiency, needle positioning, and suture placement. Less effective transfer of skill was observed in the domains of force sensitivity, robotic control, needle entry, needle driving, tissue approximation, and knot tying [24].

The transfer of technical skills across robotic platforms is sustained in all studies, and proportionate to prior robotic experience. Variations in learning curves, the usability of platforms, and unequal directions of skill transfer between platforms may exist.

Participant Reaction to Novel Robotic Systems and Perceptions of Technical Skill Transfer

The educational impact of the studies reached level one of the modified Kirkpatrick models [25]. Participants' reaction to robotic surgery transfer of skill was largely positive when describing and applying technical skills to a new console [21]. Cognitive load and difficulty ratings were inversely proportionate to the robotic experience of participants when operating a novel robotic platform [24].

Safety Metrics

Data relating to safety was measured using incidences of collisions of arms, dropped items, time instruments were out of view, and excessive force [21-24]. Occurrences were rare overall [21,22], and similar across platforms [21]. When they did occur, they were more common in the naïve cohort than those with either laparoscopic or robotic experience [22].

Kinematic Data

Expert robotic surgeons transfer technical skills with statistically significantly reduced time to complete tasks compared to novices. However, the economy of motion (instrument path length) is similar between the groups [22]. A larger master workspace area (geospatial zone of instrument control) was observed in console B regardless of the order of use in comparison to console A [21].

Discussion

Several components arise when discussing the transferability of technical skills from one robotic system to another. Firstly, it has not been previously reported if skills are transferable. Indeed, were this to be proven, the assessment environment and its translation to clinical practice must be quantified. Cross-platform training and the impact on learning curves will need to be determined to enhance training opportunities for residents and ensure satisfactory rates of skill acquisition.

Transferability of Technical Skill and Application to Clinical Practice

Novices have similar performance across consoles [21,24]. Prior robotic experience improves performance on a novel platform [22-24]. Experts on one console did not transfer all previously acquired skills immediately to new platforms [23,24]. This may be accounted for by differences in configuration such as the fine motor techniques required for the telemanipulation of instruments. Device handling may differ by three-dimensional responses of instruments, grasping strength, and torque. Decreased performance is observed in expert robotic surgeons on a novel platform in comparison to those with extensive use of the same robotic system [24]. However, the performance of expert robotic surgeons naïve to a platform appears to quickly reach the expert level on the novel system, when undergoing a structured familiarization process [23]. Clinical outcomes [26] and consensus guidelines [27] on multi-platform use support this practice [26]. Though limited in study design and number, training to competency on one robotic surgical system suggests the same level of competency on another robotic system, after a period of orientation [21-24].

High-stakes environments with non-standardized design and intermittent use of systems are not unique to robotic surgery, or indeed healthcare. Pilots operate aircraft with variations in configuration and handling, while armed forces' use of weaponry or ordnance alternates with the tactical situation. The paradigms of cross-modality training, recency requirements [28], and simulated practice for high acuity low occurrence events are evident in these disciplines. Robotic surgery technical skills appear transferable. A question remains if there is a correlation between the recency of robotic platform use and an expert’s performance and management of adverse events. Learning curves to reach proficiency on the Da Vinci robotic system have been observed [29]. Results are emerging for other platforms [30]. A direct comparison of multi-platform and single-platform experts across consoles is needed to determine the true effect on skill acquisition and maintaining competence in performance.

Training in the Multi-platform Robotic Surgery Era

The consequences of moving to a multi-platform robotic surgery training scheme are myriad. A different multi-platform and single-platform learning curve may exist [23]. Data are conflicting as to the accelerating/decelerating learning effect when transferring skills across platforms [21,23]. Educational theory would support its accelerated learning through the use of dispersed deliberative repetitive practice with variation in content [31]. It may be that the order of robotic platform use has an effect on residents’ transfer of skills [21]. In this context, the acquisition of skills [23] and skill decay with the recency of platform use need to be ascertained and plotted. If steep forgetting curves (attrition of skills) [32] with a lack of recent robotic system use exist, it will severely hinder technical skills acquisition and prolong the length of training. This must be determined to guide competency-based curriculum development and certification. 

Kinematic data can provide an objective automated assessment of performance [33,34]. Given the ongoing growth in the practice of robotic surgery, residents will be exposed to multiple platforms in clinical practice and training. The granular data available may not be suitable for objective assessments in this context. Though not statistically significant, performance times on console B were superior to console A, and yet had poorer kinematic performance [21]. Masterwork area usage and economy of motion (instrument path length) were equivalent in expert robotic surgeons and novices on a novel robotic platform. Despite this, improved competency was observed in the expert group [23]. If path length was equivalent but task completion time decreased, then instrument velocity must have been greater for the expert cohort. Therefore, the incorporation of kinematic data into assessments requires further clarification. Identifying if key metrics (e.g., velocity) predict the transfer of skills across platforms must be determined. The use of kinematic data in multi-platform training may be beneficial to direct coaching for orientation to novel consoles.

The innate ability has a predictive effect on surgical learning and proficiency [35,36]. Fine motor hobbies are indicative of performance on the Da Vinci platform [37,38]. There is no evidence pertaining to these attributes and their influence on skill transfer across platforms. Innate aptitude is used in the selection for residency. Further adoption of robotic surgery is forecasted [39]. The evolution of training to multi-platform robotic systems warrants revalidation of these quantities to ensure the continued appropriate selection of candidates who will be most suitable to complete and excel in surgical residency programs of the future.

Limitations

Inhibitory financial costs and logistical constraints are likely to reflect the scarcity of studies examining the transferability of technical skills across robotic platforms. Of those that have been conducted, they are heterogeneous in nature and have low participant numbers, often with wide variation of technical experiences. Outcome criteria and performance tasks used for assessment are not standardized. These limitations preclude further extrapolation of results and the synthesis of broader themes.

The application of these study findings to the real world is not quantified. Furthermore, all studies were conducted within a simulated environment observing a set micro-task or procedural component. Though a UVA has been validated as predictive and indicative of operative performance, its micro-task nature does not prove overall competence in transferring skill in the in-vivo operative setting. Nevertheless, preliminary clinical data comparing robotic platforms would appear to support the safety of multi-platform use [26].

## Conclusions

A dearth of validated evidence exists on the transferability of skills between robotic surgical systems. The limited publications identified suggest novices operate at a similar performance level between platforms. Experts on a prior console transfer many domains of technical skill, but still have a learning curve on the new system. Safety metrics appear comparable. To outline the next iteration of surgical training curriculums, future research must determine the factors governing the transfer of technical skills across platforms.
